# A Dog's Eye View of Morphological Diversity

**DOI:** 10.1371/journal.pbio.1000452

**Published:** 2010-08-10

**Authors:** Liza Gross

**Affiliations:** Senior Science Writer/Editor, Public Library of Science, San Francisco, California, United States of America

**Figure pbio-1000452-g001:**
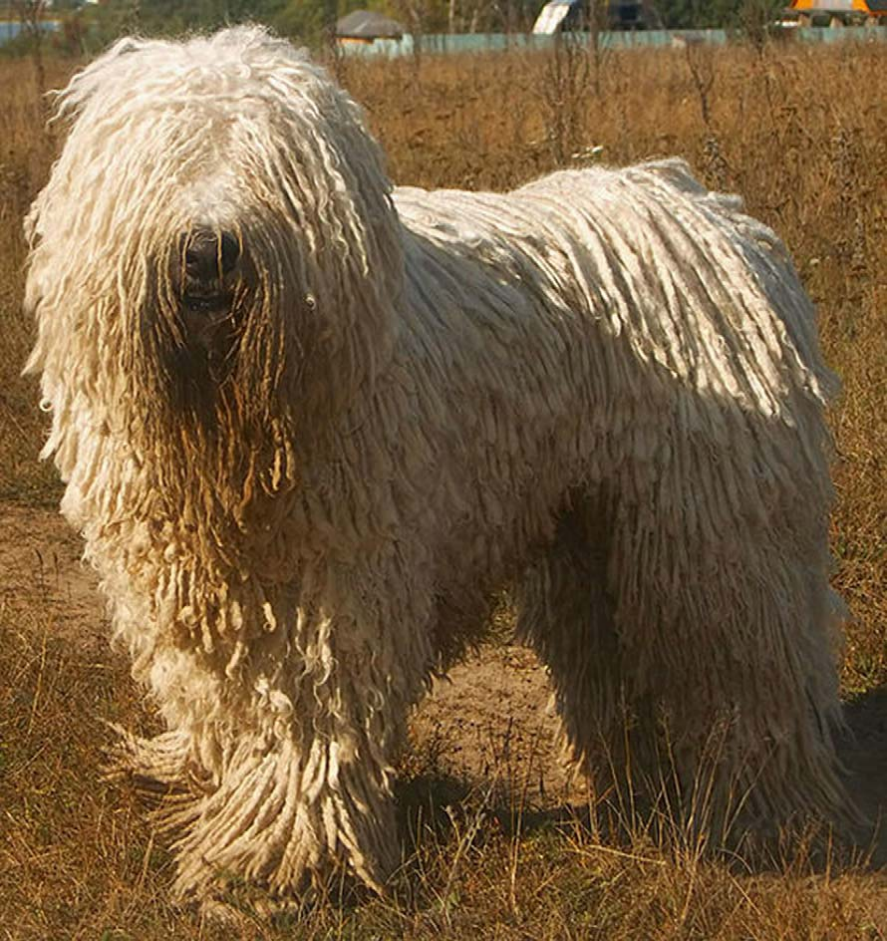
The tremendous phenotypic diversity seen in different types of pure-bred dogs, like this komondor, allows geneticists to link genomic loci with the physical expression of complex traits like hair and body size. (Image: Nikki68/Wikimedia)

[Fig pbio-1000452-g001]The history of genetic discovery offers a lesson in inspired choices. Mendel pried the principles of inheritance from the seeds, pods, and flowers of pea plants. Morgan linked trait inheritance to specific chromosomes after the unexpected appearance of a white-eyed fly among the red-eyed multitudes. McClintock showed that genes could change position on a chromosome by charting the idiosyncrasies of every leaf and kernel of her beloved maize plants.

And now Elaine Ostrander, who admits a sense of “awe” and “marvel” at her pet organism, the domestic dog, has found that some complex traits may not have such a complex genetic basis after all. Working with longtime collaborators Carlos Bustamante and Robert Wayne, Ostrander and her colleagues report that complex traits like body size and coat color may fall under the control of surprisingly few genes.

Early on, Ostrander recognized the dog's potential to undercover the genetic basis of complex traits. Morgan's white-eyed flies arose from a single-gene mutation, but most traits spring from more complex genetic interactions. Complex (or quantitative) traits are controlled by more than one gene, in combination with environmental factors, and vary by degree, with snout, for example, ranging from recessed to elongated.

Pure-bred dogs, with their storied history of intensive breeding for humans' prodigious preferences, offer geneticists a unique opportunity to link variations at a given spot in the genome (the genotype) with the physical expression (phenotype) of complex traits. From the puny pug's snub nose to the regal collie's prominent snout, the squat corgi's stubby legs to the sleek saluki's willowy limbs, the hairless chihuahua's nervous reserve to the dreadlocked komondor's fearless courage, no other land mammal approaches the dog's phenotypic diversity. Breeders maintain these archetypes by selecting for nearly every facet of a dog's being, leaving breeds with restricted gene pools and prone to genetic disease.

Still, such clear divisions among breeds help geneticists match genes with traits. Researchers have recently linked numerous gene variants and so-called quantitative trait loci (QTL)—regions of DNA associated with a trait—to several classic traits in dog breeds, including leg length, coat color, and skeletal size. It's possible that this diversity results from several QTLs with weak effects or from just a few QTLs with large effects.

To explore these possibilities and track the signs of human selection in the dog genome, Ostrander, Bustamante, and their colleagues developed a map of canine genetic diversity. Dogs, like humans, have two copies of every gene. The copies may have identical sequences (called homozygous) or different sequences (heterozygous). Using DNA from registered breeds and wild canids (915 dogs from 80 breeds, plus 83 canids, including wolves, jackals, coyotes, and feral African “village” dogs), the authors determined the sequence of over 120,000 spots in the genome likely to harbor single nucleotide polymorphisms (SNPs, pronounced “snips”)—that is, they determined which of the four DNA bases (A, C, T, and G) occupy these sites. Such single-base variations, where one dog may have an A and another a T, promise to reveal the genetic roots of morphology, behavior, and disease.

Using statistics, scientists can identify a SNP at one spot in the genome that occurs with another SNP at an adjacent location more often than one would expect by chance. The SNPs may or may not occur within a gene. The nonrandom association of SNPs (called linkage disequilibrium) and blocks of DNA can help researchers map genome regions that encode heritable traits and also provide clues to an organism's evolutionary history.

One would expect that sequences inherited from the gray wolf, the dog's evolutionary forerunner, would show up as shorter blocks of shared DNA (broken up by the random mixing of DNA from both parents over generations). Likewise, the genetic imprint of inbreeding, the lot of all official dog breeds, might be long stretches of identical sequences (referred to as “runs of homozygosity,” or ROHs).

Not surprisingly, individuals within breeds share long stretches of identical sequence (ROHs) while individuals across different breeds—and African village dogs and wolves, which mate randomly—do not. Interestingly, different breeds share many more linked loci than wolves do, supporting the notion that dogs went through a genetic bottleneck during domestication. Even so, village dogs harbor more genetic diversity than the gray wolf, perhaps because they managed to keep their populations large enough to avoid inbreeding, unlike the relentlessly persecuted wolf.

The finding that dogs from different breeds do not share large chunks of DNA, the authors explain, suggests that different breeds share few sequences inherited from their ancestors. But, they reasoned, sequences shared among breeds with similar features may well represent the genetic resources from which humans fashioned the remarkably diverse expression of these traits.

Indeed, the authors linked several shared sequences to genetic variants affecting classic morphological traits, including fur length and texture, coat color, stubby legs, snout length, and body weight. When breeders selected for variations of these traits, they unwittingly targeted certain regions of the genome, but which ones?

To find out, the authors looked for correlations between the frequency of specific genetic variations and specific phenotypes—including body size, ear type, and skull, dental, and skeletal dimensions—across 80 breeds. For body size, where dogs take the prize for biggest range among terrestrial mammals, six genomic regions stood out, including areas with genes known to influence body size. For ear type, another breed-defining trait, just one region emerged as the likely source for everything from the pharaoh hound's outsized, erect ears to the basset's low-hanging, floppy lugs. The modern mutation in this area is nearly ubiquitous in floppy-eared dogs and the region shows greatly reduced sequence diversity, both indicators of strong selection.

In nearly all the traits studied, the authors report, just a few high-impact QTLs accounted for the phenotypic variations across breeds. Interestingly, genome-wide association studies in humans suggest just the opposite: that most complex human traits fall under the control of hundreds of genes of small effect.

The patterns of linked genetic regions, with so few controlling trait diversity, indicate that breed dogs (and village dogs) went though a bottleneck at domestication, followed by another bottleneck, resulting from strong selection as humans aggressively bred dogs for whatever trait struck their fancy.

Aside from proving the dog's value as a genetic model, this study offers researchers a treasure trove of genetic data to pair genes with traits, illuminate the dog's evolution from wolf to companion, and secure its place as the geneticist's new best friend.

**Boyko AR, Quignon P, Li L, Schoenebeck J, Degenhardt JD, et al. (2010) A Simple Genetic Architecture Underlies Morphological Variation in Dogs. doi:10.1371/journal/pbio.1000451**

